# Ability to Consolidate Instances as a Proxy for the Association Among Reading, Spelling, and Math Learning Skill

**DOI:** 10.3389/fpsyg.2021.761696

**Published:** 2021-10-20

**Authors:** Chiara Valeria Marinelli, Paola Angelelli, Marialuisa Martelli, Mara Trenta, Pierluigi Zoccolotti

**Affiliations:** ^1^Department of Clinical and Experimental Medicine, University of Foggia, Foggia, Italy; ^2^Department of History, Society and Human Studies, Lab of Applied Psychology and Intervention, University of Salento, Lecce, Italy; ^3^Department of Psychology, Sapienza University of Rome, Rome, Italy; ^4^Neuropsychology Unit, IRCCS Fondazione Santa Lucia, Rome, Italy

**Keywords:** reading, spelling, Maths, automatization, learning

## Abstract

Learning skills (as well as disorders) tend to be associated; however, cognitive models typically focus either on reading, spelling or maths providing no clear basis for interpreting this phenomenon. A recent new model of learning cognitive skills proposes that the association among learning skills (and potentially the comorbidity of learning disorders) depends in part from the individual ability to consolidate instances (taken as a measure of rate of learning). We examined the performance of typically developing fifth graders over the acquisition of a novel paper-and-pencil task that could be solved based on an algorithm or, with practice, with reference to specific instances. Our aim was to establish a measure of individual rate of learning using parameters envisaged by the instance theory of automatization by Logan and correlate it to tasks requiring knowledge of individual items (e.g., spelling words with an ambiguous transcription) or tasks requiring the application of a rule or an algorithm (e.g., spelling non-words). The paper-and-pencil procedure yielded acquisition curves consistent with the predictions of the instance theory of automatization (i.e., they followed a power function fit) both at a group and an individual level. Performance in tasks requiring knowledge of individual items (such as doing tables or the retrieval of lexical representations) but not in tasks requiring the application of rules or algorithms (such as judging numerosity or spelling through sublexical mapping) was significantly predicted by the learning parameters of the individual power fits. The results support the hypothesis that an individual dimension of “ability to consolidate instances” contributes to learning skills such as reading, spelling, and maths, providing an interesting heuristic for understanding the comorbidity across learning disorders.

## Introduction

Learning disorders (such as dyslexia, dysgraphia, and dyscalculia) tend to co-occur. This phenomenon is difficult to interpret within the traditional cognitive literature as models of reading, spelling, and maths are typically distinct and offer little basis for understanding the reasons of the possible overlap between these deficits. In his seminal paper, Pennington (2006) emphasized the need to view learning disabilities as well as other developmental disorders (such as ADHD or language impairment) within a multi-factorial interpretation. Thus, different cognitive factors may contribute to the emergence of a given deficit (e.g., dyslexia) and these factors partly overlap with factors accounting for other deficits (e.g., dyscalculia). In recent years, this perspective has driven an increasing amount of research. Thus, several studies examined the co-morbidity between reading and math disorders searching for cognitive factors accounting for their comorbidity even though these studies have not yet converged on a single interpretation (Wilson et al., 2015; Slot et al., 2016; Cheng et al., 2018).

In the present study, we capitalize on previous ongoing work from our research group in which we carried out an initial study on reading, spelling, and maths learning skills in a sample of typically developing children (Zoccolotti et al., 2020a,b). As predicted within the comorbidity perspective (Pennington, 2006), we observed considerable overlap between these learning skills. Indeed, cross-analyses indicated that predictors of reading accounted for performance in calculation much better than did general cognitive predictors; furthermore, maths tests predicted quite well reading and so on (Zoccolotti et al., 2020b). Analyzing individual predictors, we observed that some predictors were specific for a single behavior (e.g., phonological tests predicted only spelling skills), others predicted different behaviors but only for a specific parameter, such as fluency but not accuracy (as in the case of RAN), and finally some variables predicted reading, spelling, and maths skills in quite similar ways.

**Figure 1:**
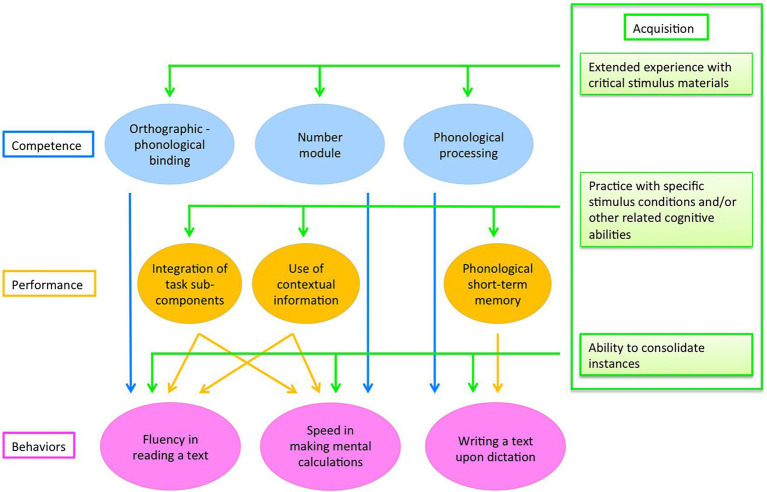
A multi-level model of learning cognitive skills. Target behaviors are expressed in terms of task-specific exemplars (Zoccolotti et al., 2020b).

To interpret this complex pattern of results, we proposed a multi-level model of learning cognitive skills (Zoccolotti et al., 2020b; see Figure 1). To this aim, we refer to the distinction between “competence” and “performance,” originally put forward by Chomsky (1966) in the discussion of language. In this context, “competence” is the general capacity to process in a given domain, while “performance” refers to the fact that a measure with a given task in a given individual is not a direct measure of competence in that domain but the result of an interaction between competence and the specific characteristics of the task. Thus, the critical difference between competence and performance is that the former is task independent, while the latter is task specific. In this perspective, all measures of a given behavior depend upon both the competence in a specific domain and the performance on the specific task. Consequently, one may assume that deficits in a specific competence (e.g., reading) will show up pervasively across different types of tasks in the domain (such as reading meaningful texts, list of words, pseudo-words.). Conversely, other deficits may be task specific to the extent in which they appear contingently to the requirements of the actual task (e.g., a child may have problems in maths under time pressure while being accurate if enough time is given), pointing to the role of “performance” components. Furthermore, a third level of explanation was posited to relate to the process of “learning” or “acquisition,” and particularly to its automatization phase. Acquisition occurs through the effect of practice: learning disorders do not refer to the inability of the child to learn to read or to do computations as much as to *the inability to do so smoothly and efficiently* (Zoccolotti et al., 2020b). Thus, children with dyslexia characteristically read in an effortful, not automatic fashion; in order to read, the child has to place all his/her cognitive resources on decoding the text with little residual ability left for comprehension.

It should be observed that practice affects behavior in different ways influencing all levels of learning skills (not just the acquisition level). Thus, practice is necessary to bring out a “competence” in reading as well as in spelling or maths. Furthermore, practice is necessary to optimize behavior in specific task conditions (“performance”), such as learning to read in a left to right manner or to write using the appropriate hand movements. However, extended practice can also influence behavior by producing automatized responses to specific target items (acquisition level). This would contribute to the ability to read (or spell) words (or make multiplications) not based on grapheme to phoneme conversion (or counting digits) but on direct obligatory memory retrieval of specific target items. Thus, through extended practice the child learns specific items (e.g., regular frequent words, but also irregular words such as “pint,” or the output of simple mathematical operations such as 3×8=24 or 5+3=8).

A theoretical formalization of the automatization process has been put forward by Logan (1988, 1992). His “*instance theory of automatization*” states that automatization is acquired through repetitive presentation of a stimulus: in this way, the “instance representation” of an individual object or event is stored in memory (“obligatory encoding”) and, the more repetitions, the more information becomes directly available (“obligatory retrieval”). The course of learning is initially fast and becomes progressively slower over target repetitions; this pace of learning is well described by a power function (as originally proposed by Newell and Rosenbloom, 1981).

Overall, the multi-level model of learning skills (Zoccolotti et al., 2020b) aims to predict both dissociations of deficits (as did previous traditional models) but also associations of deficits (i.e., comorbidity). In particular, it is assumed that independent competences are present for reading, spelling, and doing maths and that this may account for the observed dissociations among learning disorders. On the other hand, associations are expected whenever behaviors call upon the same performance factors (such as when tasks call for a speeded response or require processing contextual information; see Figure 1). Critical for the present study, it is proposed that associations among learning disorders may also be due to an acquisition factor and particularly to the “*ability to consolidate instances*” which is responsible for automatized behavior (see Figure 1). Accordingly, some children may have a low ability to consolidate instances (automatize) and this may influence their performance in reading (by limiting their ability to form lexical entries) as well as in spelling (again, limiting lexical acquisition) and doing maths (dampening the ability to acquire arithmetic facts). In this view, the ability to automatize is a factor that contributes to efficient performance across different domains. Thus, poor ability in forming instances does not make the behavior impossible but rather dampens fast and fluid reading, efficient spelling, and fast and efficient calculation (Zoccolotti et al., 2021). Indeed, children with dyslexia are not unable to read, but their reading is cumbersome, inefficient, and ultimately tiring, characteristics which indicate a controlled, voluntary mode of processing; by contrast, typically developing peers are characterized by smooth and efficient decoding which marks their pre-attentive, automatic processing (Schneider and Chein, 2003).

Within the multi-level model of learning cognitive skills (Zoccolotti et al., 2020b), a number of predictions follow from this hypothesis on automatization. First, one would expect lack of automaticity to be associated across reading, spelling, and maths. Consistently, it has been reported that adults with dyslexia were defective in their ability to retrieve arithmetic facts, although their numerical representations were spared (De Smedt and Boets, 2010). Second, one would predict that failures in activating lexical entries should be item specific. Angelelli et al. (2010a) examined the consistency of a lexical deficit between a reading (orthographic decision) and a spelling task. Fifth grade children with dyslexia failed to judge the orthographic correctness of the very same words with irregular transcription which they failed to spell. Thus, their lexical deficit was item specific but consistent across reading and writing. Third, one would expect that deficits due to a defective ability to consolidate instances should emerge more clearly late in the course of development, when the typically developing children have consolidated their knowledge of many items allowing fast and smooth reading (spelling or doing maths). Findings along this line have been reported in terms of spelling skills by Angelelli et al. (2010b). Thus, while in third grade, the spelling deficit was generalized encompassing all stimulus categories, in fifth grade errors for spelling words with unpredictable transcription were on the foreground, indicating a prevalent lexical impairment. A prevalent lexical impairment and a deficit in the expansion of the orthographic lexicon in children with developmental dyslexia were also supported by the longitudinal study of Marinelli et al. (2017). Finally, the model predicts that the ability to retrieve individual instances would be independent of the core competence in a given learning skill (i.e., either reading, spelling or maths). Consistently, it has being recently reported that, in spite of their item-based lexical deficit in both reading and spelling, children with dyslexia showed appropriate sensitivity to the distributional information of sound-spelling mappings at sub-lexical level (Marinelli et al., 2017, 2021; Angelelli et al., 2018). Overall, there are experimental data supporting the idea that at least part of the deficits in reading, spelling or maths may be ascribed to a general, cross-domain defect in consolidating individual instances.

Still, it is difficult to use the evidence available in the literature to fully evaluate this hypothesis. On the one hand, data on lexical orthographic knowledge or knowledge of arithmetic facts tell us something about the outcome of the process, but they are not informative about the developmental trajectory of how children have reached a given level of performance. On the other hand, a number of studies have compared children with learning disorders and controls during the course of acquisition. In particular, various studies have examined how children with dyslexia learn pseudo-words over a number of repetitions (Martens and de Jong, 2008; Pontillo et al., 2014; Suárez-Coalla et al., 2014; Kwok and Ellis, 2015). Most of these studies have reported that children with dyslexia learn less rapidly than controls and that, by the end of the training period, they typically maintain a strong sensitivity to the influence of stimulus length (Martens and de Jong, 2008; Pontillo et al., 2014; Suárez-Coalla et al., 2014; Kwok and Ellis, 2015). Thus, these studies are consistent with the idea that children with dyslexia are less efficient in learning and forming new representations of individual items (or lexical entries). However, most of these studies are focused on a single behavior (i.e., reading) and as such are not informative as to the breath of the influence of this differential learning across behaviors. Nicolson and Fawcett (2000) examined long-term acquisition of children with dyslexia in a more general perspective. In two studies, they examined the performance of a group of dyslexic adolescents and a matched group of typically developing controls on long-term training of two different tasks (a simulated pacman game and a choice reaction time task). In the pacman game, the dyslexic adolescents showed lower initial performance and, while they improved over time, the general performance differences were maintained by the end of the training. Similar results were present in the choice reaction time task. Nicolson and Fawcett (2000) interpret this pattern of findings as consistent with the hypothesis that dyslexia would be linked to a deficit in automatization possibly associated with cerebellar dysfunctioning.

As stated above, the multi-level model of learning skills (Zoccolotti et al., 2020b) proposes that a low ability to consolidate instances represents a domain-independent factor which may account for a sizeable part of the association among different learning skills (and potentially for the comorbidity among different developmental disorders). To provide for a sensitive test of this hypothesis in the present study, we examined the ability of an unselected group of children to learn a novel task allowing to examine the typical shift with practice from an algorithm-based to an instance-based performance. The experiment was modeled after the instance theory of automatization put forward by Logan (1988, 1992). Accordingly, one expects that, with practice, performance (in terms of time) changes following a power function, i.e., improvements in performance are greatest in the first trials and become progressively smaller with continuing practice. While most studies based on this model use reaction time measures, in order to simplify the paradigm for the use with children we devised a new paper-and-pencil test. This allowed us to test a sufficiently large sample of participants. We reasoned that, if the curves of learning follow the predicted power law of practice (Logan, 1992), this would allow establishing individual performance in terms of a number of critical parameters: the scaling parameter a (i.e., the asymptote, reflecting an irreducible limit on performance); the scaling parameter b (i.e., the difference between initial and asymptotic performance); and the exponent c (which determines the shape of the function).

We hypothesized that the individual ability to consolidate instances with learning opportunities of a child would be correlated with his/her ability in tasks that call for the specific knowledge of individual items, such as spelling or making an orthographic decision on a word with ambiguous transcription or retrieving arithmetic facts. Critically for the model presented in Figure 1, this association should hold irrespective of behavior, i.e., in reading, spelling as well as maths. Conversely, we did not expect that the individual ability to consolidate instances would be associated with tasks that call into action the application of algorithms (such as spelling non-words) or the abstract ability to represent number quantities. It must also be acknowledged that, in several tasks, performance may be aided by knowledge of individual items though it is ideally possible to carry out the task also without such reference (i.e., solely based on the application of rules or algorithms). For example, this is the case of reading or spelling of regular words or carrying out mental or written calculations. Thus, a child may read (or spell) a regular word either with reference to the grapheme to phoneme conversion rules or by referring to the lexicon. In calculation, the child may use algorithm-based procedures but may also speed up his/her performance by using knowledge about specific arithmetic facts.

Operationally, we examined the performance of a sample of fifth grade typically developing children over the acquisition of a novel task that could be solved with reference to an algorithm or, with practice, with reference to specific instances. Our aim was to establish measures of their individual rate of learning (i.e., their ability to consolidate instances) using the parameters envisaged by the instance theory of automatization (Logan, 1988, 1992). Then, we examined if such learning ability would predict performance in tasks that require knowledge of individual items (such as spelling words with an ambiguous transcription) as well as to measures that do not call for the knowledge of individual items (such as spelling non-words). We expected that individual rate of learning should be associated with the former but not to the latter. For exploratory reasons, we also included tasks for which no explicit predictions could be advanced, i.e., tasks that can be solved either by knowledge of individual items or by the application of algorithm-based rules (such as spelling regular words or making written calculations). Finally, as a further control we also included tasks mapping domain-general skills (i.e., non-verbal intelligence and short-term memory) for which we expected no specific relationship with the rate of learning dimension.

## Materials and Methods

### Sample

A total of 140 children accepted to participate in the experiment. Three children with an impaired performance on the Raven’s Coloured Progressive Matrices (CPM; i.e., 2 standard deviation below the according to Italian normative values, Pruneti et al., 1996) were excluded from the sample. Then, participants were 137 Italian children (82M, 55F, mean age=10.36, SD=0.60) attending fifth grade schools in areas of Lecce and Roma characterized by a middle-class socio-educational conditions. As described in detail below, we focused our analyses only on the children whose performance on our experimental task proved reliable, a procedure which led to exclude additional 12 children (ca 8.8% of the original sample). Thus, the subsample analyzed in the present study eventually included 125 Italian children (78M, 47F, mean age=10.34, SD=0.61). The mean z score in the CPM test was about zero for the whole sample of 137 children (Mean=0.20, SD=0.89) as well as for the subsample of 125 children (Mean=0.20, SD=0.91).

Parents were informed about the screening activities and authorized their child’s participation by signing the appropriate informed consent paperwork. The study was carried out in accordance with the principles of the Declaration of Helsinki and was approved by the school authorities.

Children were tested with several tests evaluating mathematical, reading, and spelling skills, as well as domain-general skills and the instances acquisition ability. A description of these various tests used follows.

### Tests

#### Reading Assessment

##### MT Reading Test

The participant must read aloud a passage within a 4-min time limit (Cornoldi and Colpo, 1998). Speed (seconds per number of syllables read) and accuracy (number of errors, adjusted for the amount of text read) were scored.

##### One Minute Reading Test

The test evaluates together speed and accuracy in reading aloud words (Turner, 1987). We used the Italian version of the test (Marinelli et al., in preparation). It consists of a matrix of 158 short (5-letter) bi-syllabic low-frequency words (mean=15.54, SD=6.44; range 6–30, according to the children’s word frequency database, Marconi et al., 1993). Words are presented simultaneously on a grid format as in a RAN matrix. Children have to read aloud as many words as possible processing from left to right within the time limit of 1min. The score was the number of words correctly read in one minute.

##### Orthographic Decision Test

In this test, children have to judge the orthographic correctness of 72 words with inconsistent spellings due to the presence of a phonemic segment with two homophonic transcriptions (only one of which is orthographically correct) and, for control, of 36 regular words (i.e., not containing any inconsistently spelled phonemic segment) (Marinelli et al., 2017). Half of each experimental set was made of high-frequency words (mean=242.6, SD=385) and half of low-frequency words (mean=5.6, SD=5) according to Marconi et al.’s (1993) database.

A pseudo-word (composed of legal letter sequences) was created for each stimulus. Pseudo-words derived from inconsistently spelled words were pseudo-homophones (i.e., they resulted in a string that could be read as homophonous to the target; e.g., ^*^SQUOLA derived from SCUOLA, school). Thus, they can be detected only by relying on the lexical procedure. Pseudo-words derived from regular words resulted in strings that were non-homophonic because of the substitution or permutation of graphemes (e.g., DENORO derived from DENARO, money). They can be detected through either the lexical or the sub-lexical procedure. The accuracy is scored (for more details see Marinelli et al., 2017).

#### Spelling Assessment

##### Single Word and Pseudo-Word Dictation Test (DDO-2 Short Version)

The test is composed of four sections: Section A (*N*=24): Words with full one-sound-to-one-letter correspondence: Section B (*N*=6): Words requiring the application of context-sensitive sound-to-spelling rules; Section C (*N*=15): Words with unpredictable phonology-to-orthography mapping (i.e., ambiguous words; e.g., /kwo/in/kwota/, share): QUOTA and not ^*^CUOTA) and therefore writable correctly only using the lexical way; and Section D (*N*=15): Pseudo-words with one-sound-to-one-letter correspondence (Angelelli et al., 2016).

Words and non-words are presented in separate lists and in randomized order. The examiner reads each item aloud without emphasizing the presence of difficulties; the children are asked to repeat it (to make sure they have understood it) and afterward to spell the stimulus. The number of spelled correctly items in each section is computed.

##### “Nonna Concetta” Passage Dictation Test

The task is a spelling to dictation test, consisting in a meaningful passage that includes words with regular and unpredictable spelling, tapping the efficiency of both lexical and non-lexical spelling procedures (Marinelli et al., 2016). The experimenter reads the meaningful passage, following the pauses established by the test. The child has to spell the text on a white paper. The scoring is made by calculating the total number of elements correctly spelled.

#### Mathematical Skills

##### Written Arithmetic Calculations Test (From the AC-MT Battery)

This test assesses child’s ability to perform 8 written computational operations (two calculations for each of the four basic number operations: addition, subtraction, multiplication, and division; Cornoldi et al., 2002). The number length varies from 3 to 5 digits and sometimes includes decimals. One point score is given for every correct calculation.

##### Number Ordering Test (From the AC-MT Battery)

This task assesses semantics of numbers (Cornoldi et al., 2002). Ten series of four numbers are presented, and the child must be able to place them in the correct order (5 series from the largest to the smallest; 5series from the smallest to the largest). Accuracy is recorded.

##### Dictation of Numbers Test (From the AC-MT Battery)

This task assesses students’ ability to activate lexical retrieval as well to elaborate the syntactic structure of number. Students listen 8 numbers over a thousand, and they have to spell them (Cornoldi et al., 2002). Accuracy is scored.

##### Judgment of Number Magnitude Test (From the AC-MT Battery)

This task assesses students’ ability to understand semantics and syntactic proprieties of numbers, asking them to indicate the bigger one of a couple of numbers (Cornoldi et al., 2002). Accuracy is scored for a total of 6 items.

##### Backward Counting Test (From the AC-MT 6–11 Battery)

The test assesses knowledge of the number line (Cornoldi et al., 2002). The child has to count backwards from 100 to 50 as rapidly and accurately as possible. Every interruption of the sequence is evaluated as error. The sum of correct numbers reported are scored.

##### Arabic Number Reading Test (From the Developmental Dyscalculia Battery, DDB)

The child has to read a list of 16 numbers (from 3 to 6 digits) aloud (Biancardi et al., 2004), without time constraints. The digit “0” is often present (e.g., “20,056” or “4,080”), in order to evaluate also children’s ability to process implicit numbers. The time to complete the task and the number of correct responses is scored. However, only accuracy was entered into the analyses.

##### Transformation of Numbers Into Digits Test (From the AC-MT Battery)

The test investigates syntactic knowledge about the positional value of the digits: 6 numbers are presented with mixed units, tens, hundreds, thousands, tenths and hundredths and the child is asked to rewrite the corresponding number (for example: 6 tens 8 hundredths 2units 0 tenths, and 5 hundreds correspond to the number 562,08; Cornoldi et al., 2002). Accuracy is scored.

##### Arithmetical Facts Test (From the AC-MT Battery)

This task investigates if children have stored arithmetical facts and are able to automatically retrieve the results of basic and simple operations from the memory (Cornoldi et al., 2002). Children are asked to recall 12 arithmetic facts, each within a maximum of 5s. Accuracy is scored. Responses given beyond 5s are considered errors, because are not retrieved automatically from memory as arithmetic facts.

##### Additions and Subtractions Within “10” Test (From DDB)

The child must say within the time limit of 2s the results of 8 additions and 8 subtractions within “10,” and thus quickly solvable with the retrieval of the arithmetic facts from memory (e.g., 4+2=?, 3–1=?; Biancardi et al., 2004). Hesitations (silent pauses longer than 2s) or responses beyond the time limits are considered invalid responses. The number of correct responses (within the 2s time limit) is scored.

##### Multiplications Test (From DDB)

The child must say the result of sixteen multiplications (for example 3 times 8, 9 times 5, etc.) as rapidly as possible (Biancardi et al., 2004). Hesitations (silent pauses longer than 2s), responses beyond the time limit or based on the use of a times table are considered invalid responses. The number of correct responses is scored.

##### Times Table in Series Test (From DDB)

The child must report the times tables of 4 and 7 (i.e., 4, 8, 12….; 7, 14, 21…) as rapidly as possible (Biancardi et al., 2004). Hesitations (silent pauses longer than 2s) are considered as invalid responses. The number of correct responses is scored, with a maximum of 20.

##### Computation Strategies Test (From the AC-MT 11–14 Battery)

Written calculations are printed on a sheet of paper, and the result of each calculation is shown along with the calculation (Cornoldi and Cazzola, 2003). Besides each complete calculation, there is a similar calculation to be computed; this latter calculation may differ from the adjacent one by inversion of the terms, increase in one of the terms by addition of a unit (or multiplication by tens), substitution of one of the terms with the result, and so on. Thus, the child can determine the result of these operations without actually calculating them but reasoning on the base of the similar complete calculations shown beside. The child is requested to perform rapidly (with an overall time constraint of 2min) as well as accurately over a total of 16 trials. The number of computations performed correctly within the time limit was scored.

#### General Cognitive Skills

##### Raven’s Coloured Progressive Matrices

This test evaluates non-verbal intelligence. The number of correct responses is scored (Raven, 1965).

##### Forward and Backward Span Of Numbers (From The Bvn Battery)

The forward task requires the immediate serial recall of a sequence of digits (Bisiacchi et al., 2005; verbal short-term memory). The span corresponds to the last length for which at least two sequences were correctly recalled. In the backward task, the child has to recall each sequence in backward order. The forward and backward spans are measured.

#### Experimental Test

**Figure 2:**
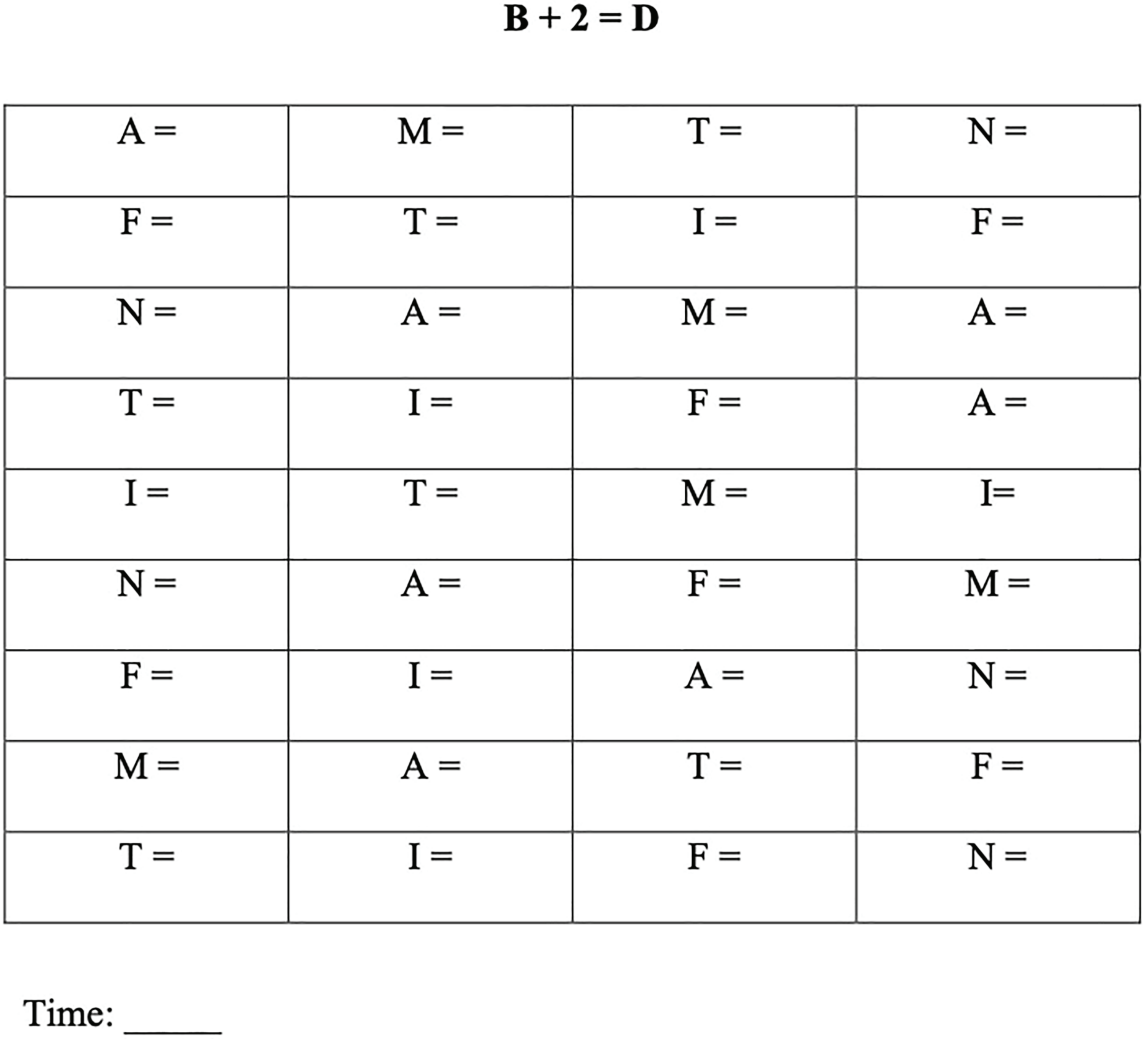
Example of a matrix of the experimental task. The rule is presented on top of the matrix.

The experimental test consists of a paper-and-pencil test, administered individually, which evaluates the learning of an invented rule (presumably never applied before by children). The stimulus features a matrix of 36 letters (with six target letters presented six times each), and the child is asked to write for each letter another letter applying the rule: letter +2 positions ahead in the alphabet=?. Thus, the task consists in advancing by two positions with respect to the starting letter written on the sheet, writing the corresponding letter next to it. An example of such a matrix is presented in Figure 2. Letters were arranged in 4×9 matrices. As shown in the figure, an example of the rule to be applied is shown at the top of the matrix (B+2=D) and is therefore always available to the child. A total of 22 matrices were devised. In the first 20 matrices (A1 to A20), the target stimuli inserted within each matrix were always the same letters (A, M, T, N, F, and I) but displayed in a different order across matrices. Two additional matrices (B1 and B2) contained different stimuli (U, C, R, E, Q, and L) and were used to examine the degree of generalization of learning to stimuli not subjected to exercise.

After explaining the instructions for the task to the children, they were given a practice matrix containing 8 letters (not used in the actual test) which was used to make sure that the child understood the instructions well. Then, the participants were presented with the series of 22 matrices, whose administration was organized in two consecutive days. In the first day, the child was given matrices from A1 to A10 and, on the second day, matrices from A11 to A20 as well as matrices B1 and B2. The test was administered individually. Children were instructed to go as fast as possible but trying to be correct. They were also informed that it was not possible to go back and correct. For each matrix, overall time (in sec.) and number of errors were measured.

### Procedure

Children were tested individually in a quiet room in their school in two consecutive days.

### Data Analysis

The Logan’s model (1988, 1992) hypothesizes that time to perform a visuo-motor task, such as the one included in our study, follows a power function as a function of practice:


(1) $$Τ=a+b{Ν}^{-c}$$

In equation 1, T indicates time, a is a scaling parameter indicating the asymptote, which reflects an irreducible limit on performance, b is a scaling parameter indicating the difference between initial and asymptotic performance, c indicates the exponent with higher values indicating steeper rates of learning and Ν is the amount of practice.

We initially used equation 1 to model the individual data with least squares method to test whether performance improved as a function of practice following a power function in compliance with Logan’s model. The asymptote a was constrained not to be lower than the minimum time spent by each observer in completing the matrices independently from the session number.

In order to evaluate the specific hypotheses of the study, we estimated the individual three main parameters: the scaling parameters a and b and the exponent. We considered for each fit the R^2^, i.e., the variance explained; higher values indicate better fits. As presented in detail in the Results section, the power fit for the total sample was quite good confirming the efficacy of the paradigm. Individual power fits were generally good, but a number of children showed somewhat irregular learning curves and accordingly had low individual R^2^ values. To use individual data, we adopted an arbitrary cut-off of *R*^2^>0.30. In this way, the data of 125 children out of the 137 tested (91.2%) could be used for further analyses.

To test our hypotheses, we used the learning parameters (a, b, and c) of each child with a curve with *R*^2^>0.30 as predictors of the performance in the various reading, spelling, calculation, and control tests. To this aim, we calculated separate multiple regression analyses (Enter method) using the performance in each test as dependent measure and the parameters of the power fit as the predictors. Our hypotheses concern the relationships between parameters of the individual power functions and the performance in tasks that require reference to individual instances but not to tasks that call for the application of an algorithm.

The effectiveness of the learning and the subsequent fall in the test in which the stimuli are modified was also evaluated with ANOVAs for repeated measurements (described more analytically in the results).

## Results

### Learning Effects in the Experimental Task

**Figure 3:**
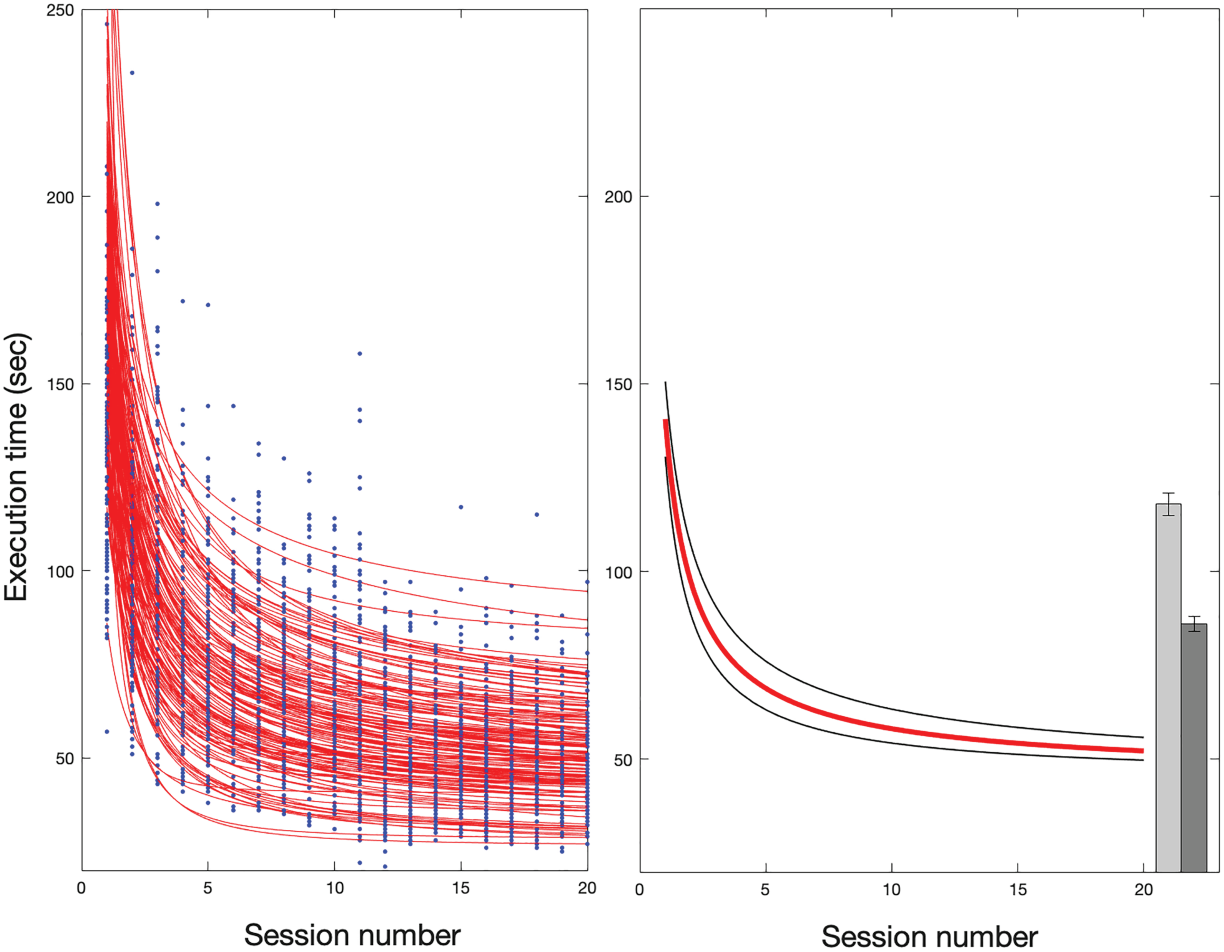
Learning trend in the experimental task. The panel on the left shows the individual fits obtained by the entire sample of 137 children. Twelve fits showed an *R*^2^<0.3, and the data were excluded from further analyses. The panel on the right shows the fit applied to the median of the data of the remaining sample of 125 children (red solid line) with an *R*^2^=0.95 and the 95% confidence intervals (black lines). The bars on the far right show the medians and standard deviations of the two retest conditions (B1=118, SD=32; B2=89, SD=24).

Figure 3 (left) shows the learning curves obtained by the sample of 137 children. The results indicate that all children reduced their time to solve the 20 matrices (i.e., from A1 to A20), with their improvement closely following a power function. In some cases, the goodness of the fit was low (*R*^2^<0.3); thus, the data of twelve children have been excluded from further analyses. The remaining sample of 125 children showed a time reduction according to a power function with a median *R*^2^=0.68 (range 0.3–0.93).

Figure 3 (right) shows the fit applied to the median of the data of the subgroup of children. Execution time reduced with practice according to the power law (*R*^2^=0.95) with the following global parameters:


$$T=45+95.6^{-0.86}$$

The figure also shows that mean performance markedly slowed down when a matrix with new items (B1, light grey bar) was presented, highlighting the specificity of instance learning. Note, however, that performance again appreciably improved at the second presentation of this new matrix (B2, dark grey bar).

**Figure 4:**
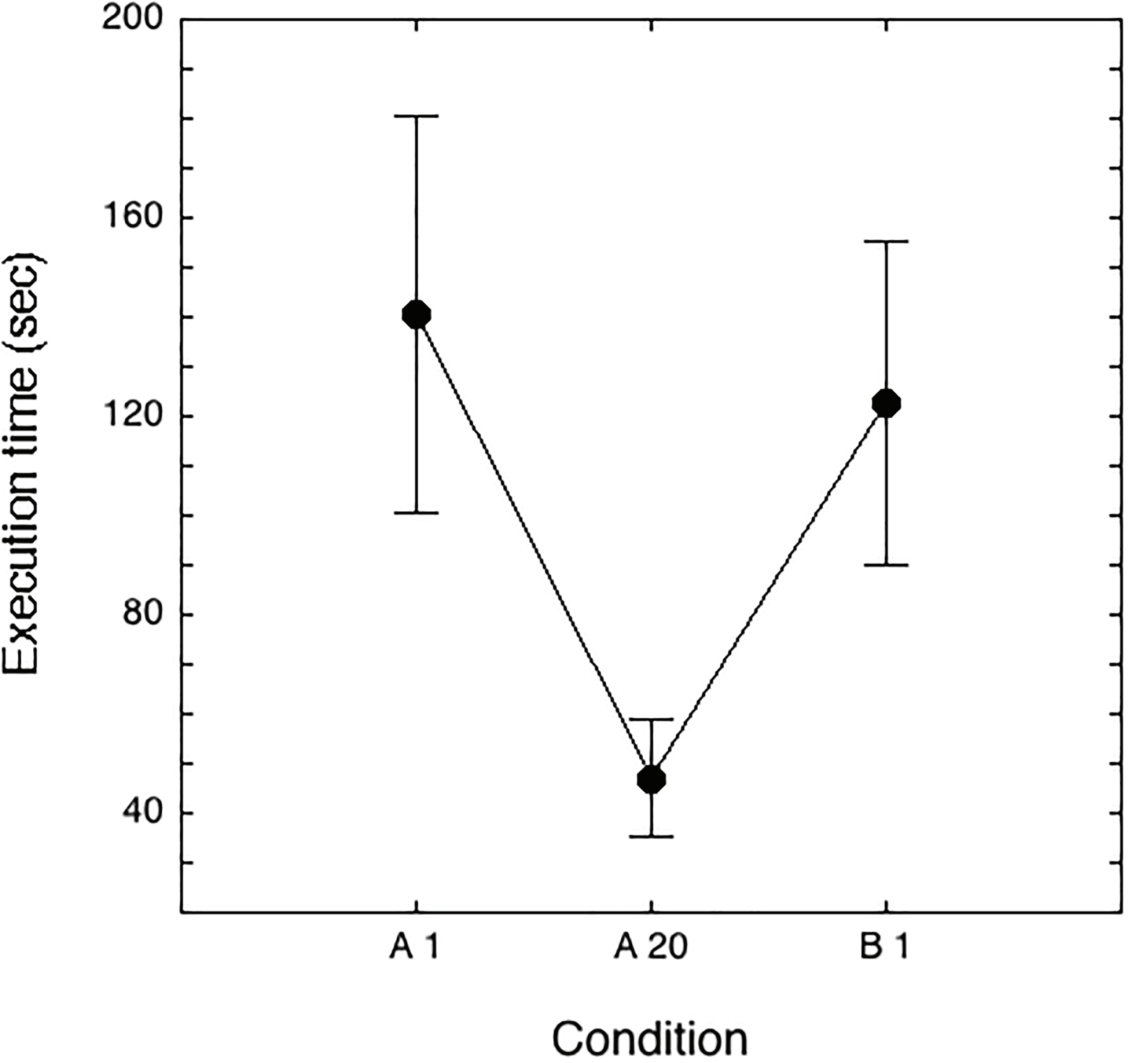
Mean time spent (seconds) and SDs in the experimental test in the A1 and A20 matrices, containing the same stimuli, and in the B1 matrix, containing new stimuli.

The effects of learning across experimental trials were also investigated with ANOVAs for repeated measurements separately for response times and accuracy. As far as response times, an ANOVA with repetition indicated a significant learning effect across the 20 repetitions (*F*_(19, 2,356)_=361.21; *p*<0.0001). In a different analysis, we compared the first presentation (A1) with the last one (A20) and the first presentation with new stimuli (B1; see Figure 4). The condition effect was highly significant (*F*_(2, 248)_=484.74; *p*<0.0001), indicating a significant decrease in times with practice (of about 94s., *p*<0.0001, Tukey’s test) and a significant increase in times in the condition with new stimuli (B1) with respect to the A20 presentation (of about 75s., *p*<0.0001, Tukey’s test); performance in the B1 presentation (122s, SD=32s) was much slower than the performance at the A20 matrix (47s, SD=12s), although slightly faster than in the A1 (141s, SD=40s) presentation (of about 19s., *p*<0.0001, Tukey’s test).

As for accuracy, the results showed a significant effect of learning across the 20 presentations (*F*_(19, 2,356)_=3.78; *p*<0.0001). A significant effect also emerged in the analysis that compared the A1, A20, and B1 presentations (*F*_(2, 248)_=6.16; *p*<0.01): errors decreased from 2.52% in A1 to 1.61% in A20 (*p*=0.09) for increasing again to 3.14% in the B1 matrix (with respect to the A20 matrix, *p*<0.001); accuracy in performing the B1 matrix was not significantly different from the A1 matrix.

### Relationship Between the Performance in the Experimental Task and Other Reading, Spelling, Calculation, and Control Tests

The multiple regression results are presented in Table 1. For the sake of clarity, we present the different multiple regression analyses according to the different learning domains (reading, spelling, maths, and control tests). Furthermore, we separately group the tasks for which a relationship with performance in the experimental tasks is expected, those for which no relationship is expected, those for which the prediction is uncertain and finally the control tasks.

**Table 1:** Each line of the table reports the results of a separate multiple regression analysis.

	Full model		a (scaling parameter)		b (scaling parameter)		c (exponent)	
	*R* ^2^	*F*	beta	*t*	beta	*t*	beta	*t*
Cases in which a correlation is predicted								
*Reading*								
Orthographic Decision	**0.107**	**4.84** ^**^	**0.17**	**2.00** ^*^	**0.25**	**2.73** ^**^	**0.13**	**1.42**
*Spelling*								
DDO-2: Words with unpredictable mapping	**0.093**	**4.13** ^**^	**−0.22**	**−2.44** ^*^	**−0.18**	**−1.94** ^*^	**0.04**	**0.39**
*Maths*								
Arithmetic Facts	**0.107**	**4.85** ^**^	**0.21**	**2.44** ^*^	**0.21**	**2.35** ^*^	**−0.03**	**−0.31**
Times Table in Series	**0.086**	**3.80** ^*^	**−0.14**	**−1.63**	**−0.23**	**−2.47** ^**^	**0.03**	**0.37**
Multiplications	**0.131**	**6.08** ^***^	**−0.25**	**−2.9** ^**^	**−0.23**	**−2.58** ^**^	**−0.04**	**−0.41**
Additions and Subtractions within “10”	**0.071**	**3.08** ^*^	**−0.10**	**−1.11**	**−0.24**	**−2.57** ^**^	**−0.09**	**−0.94**
Cases in which a correlation is NOT predicted								
*Spelling*								
DDO-2: Pseudo-words	0.032	1.31	−0.12	−1.37	−0.11	−1.17	−0.01	−0.08
*Maths*								
Judgment of Number	0.036	1.52	0.08	0.92	−0.19	−1.99^*^	−0.09	−1.02
Transformation into Digits	0.042	1.77	0.07	0.75	0.05	0.56	−0.17	−1.88
Number Order	0.021	0.85	−0.08	−0.84	−0.10	−1.06	−0.07	−0.79
Dictation of Numbers	0.006	0.15	−0.06	−0.51	0.00	0.01	−0.04	−0.38
Computation Strategies test	0.064	1.75	−0.20	−1.78	−0.13	−1.11	−0.03	−0.28
Cases in which prediction is uncertain								
*Reading*								
MT accuracy	0.056	2.37	0.13	1.42	0.14	1.53	−0.09	−0.99
MT speed	**0.132**	**6.13** ^***^	**−0.21**	**−2.44** ^*^	**−0.25**	**−2.86** ^**^	**−0.15**	**−1.68**
One-Minute test	**0.090**	**3.51** ^*^	**−0.24**	**−2.59** ^**^	**−0.10**	**−1.07**	**0.10**	**1.02**
*Spelling*								
“Nonna Concetta” dictation task	**0.125**	**5.74** ^***^	**−0.17**	**−2.02** ^*^	**−0.23**	**−2.63** ^**^	**0.12**	**1.35**
DDO-2: Regular words	0.056	2.39	0.00	0.03	−0.23	−2.45^*^	−0.15	−1.60
DDO-2: Words with context-sensitive rules	0.024	0.99	−0.14	−1.50	−0.04	−0.46	−0.04	−0.44
DDO-2: Total accuracy	**0.090**	**4.01** ^**^	**−0.19**	**−2.12** ^*^	**−0.21**	**−2.33** ^*^	**−0.03**	**−0.35**
*Maths*								
Written Arithmetic Calculations	0.026	1.09	−0.02	−0.23	−0.16	−1.72	−0.03	−0.33
Arabic Number Reading (total correct)	**0.070**	**3.04** ^*^	**−0.19**	**−2.11** ^*^	**−0.10**	**−1.12**	**0.12**	**1.32**
Arabic Number Reading (tot. seconds)	0.018	0.74	0.13	1.41	−0.03	−0.34	0.03	0.28
*Control tests*								
Raven Matrices	0.015	0.62	−0.01	−0.06	−0.12	−1.23	0.02	0.21
Digit Span forward	0.005	0.09	0.07	0.48	−0.04	−0.30	−0.03	−0.19
Digit Span back	0.092	1.82	−0.01	−0.07	−0.25	−1.83	−0.21	−1.53

*In all cases, the dependent measure is the task in the left column and the predictors entered in the analysis were the parameters of the individuals power fits. For each model, the overall value for R^2^ (and the related F value), the betas connected to the scaling parameter a (asymptotic performance), b (the difference between initial and asymptotic performance), and c (the exponent of the power function) as well as the related Student t values are reported. Significance of F and Student’s t values are indicated by asterisks. Results indicating overall significant predictions are presented in bold. For the organization of the table as a function of the hypotheses of the study see main text*.

* *p<0.05.;*

** *p<0.01 and*

*** *p<0.001*

Inspection of the table indicates that all regression analyses for which we expected a significant relationship were significant (with overall R^2^ ranging from 0.071 to 0.131). As for the contribution of different parameters in the power fits in the experimental task, the scaling parameters a (asymptotic performance) and b (difference between initial and asymptotic performance), but not the exponent c, significantly predicted the reading performance in the Orthographic Decision test (*p*<0.01) and the spelling performance on words with unpredictable transcription (*p*<0.01). The pattern of findings was similar for the maths tasks (Arithmetic Facts, Times table in series, Multiplications, Additions, and Subtractions within “10”). The scaling parameter b significantly entered in all analyses, while the scaling parameter a entered in the analyses on Arithmetic facts and Multiplications but not the other two; the exponent c did not enter significantly in any of these regression analyses.

In the case of tasks for which a relationship was not predicted, none of the multiple regression analyses were significant, as expected (the overall R^2^ ranging from 0.006 to 0.064). In one case (Judgment of Numbers), the scaling parameter b was significant but in the context of an overall insignificant prediction.

As anticipated, results were more scattered in the case of tasks for which the prediction was uncertain. In the case of reading tasks, the regression analyses were significant in the case of reading speed (MT test) and for the One-Minute test; for spelling, an overall significant prediction was present for the “Nonna Concetta” dictation task and for the total accuracy of the DDO-2 test; finally, for maths tests, the regression was significant in the case of the total correct score for Arabic Number Reading. In all these cases, the scaling parameter a significantly contributed to the multiple regression; the scaling parameter b contributed to all analyses expect the One-Minute test and the Arabic Number Reading; the exponent c did not enter significantly in any of these regression analyses. In one case (DDO-2 test: regular words), the scaling parameter b was significant but in the context of an overall insignificant prediction.

Finally, none of the models with the control tests (Raven Matrices and Digit span) proved significant (with overall *R*^2^ ranging from 0.005 to 0.092). Furthermore, none of the individual parameters showed a significant contribution.

## Discussion

The results indicate that the paper-and-pencil procedure yielded clear acquisition curves quite consistent with the predictions of the instance theory of automatization by Logan (1988, 1992). Children improved their speed in performing the experimental task across the twenty repetitions given, and their rate of improvement closely followed a power function fit, as anticipated by the theory. When matrices of new stimuli were presented, performance slowed substantially although not quite as to the original level. This pattern is predicted by the instance theory by Logan (1988, 1992) and indicates that the automatization of response is closely associated with learning of individual items (or instances).

Most children showed acquisition curves with good individual fits, and it was possible to submit to regression analyses individual values from 125 out of the total 137 children examined (91%). Therefore, it appears that the paradigm used was sufficiently sensitive and reliable to allow examining the curve parameters also at an individual level.

Results from the multiple regression analyses gave some support to the hypotheses we put forward. Children with higher performance improvement with practice (i.e., with higher b scaling values) and lower asymptotic performance (i.e., lower scaling value a) tended to have better performance in tasks in which the knowledge of individual items is specifically required during acquisition. This was the case of recalling arithmetic facts or making multiplications, additions, and subtractions without the aid of algorithms. The scaling parameters of the power fits were also associated with the performance in spelling words with an ambiguous transcription in spelling words that require to use the lexical route in spelling. Finally, they were associated with the performance in making orthographic judgments on words with ambiguous transcription. Therefore, consistent with the hypotheses, the scaling parameters of the power fits significantly contributed to models across domains, i.e., for maths, reading, and spelling tasks. Note that the exponent c did not enter as a significant predictor in any of the multiple regression analyses. Thus, it is not the shape of the curve to be critical as much as the actual change of performance (as assessed by scaling parameter b) and, in some cases, the asymptotic value reached by the child (as assessed by scaling parameter a).

In the model presented in Figure 1, the individual ability to consolidate instances is considered as an across-domain skill which favors performance whenever reference to individually learnt items (or instances) have to be recalled to efficiently perform a task. Conversely, a low ability in consolidating instances is expected to contribute to learning disorders in a way that is not domain specific, i.e., it may help accounting for the presence of co-morbidities across learning disorders. By and large, the present results were in line with these predictions.

We also predicted that individual learning rate would not be associated with tasks in which application of rules or algorithms is required and no reference to previous knowledge of individual instances can be used. For all the tasks considered in this category, no overall significant model was found as predicted. Consistently with the hypotheses, no effect was also present for control tasks, mapping non-verbal intelligence, and short-term memory.

For exploratory purposes, we also correlated individual learning rate with performance on standard clinical tests, such as reading a text passage or performing written calculations. In this case, it is difficult to anticipate predictions as performance in these tests typically calls for both the ability to activate instances (such as strategically using arithmetic facts to solve a complex calculation) and that of applying rules or algorithms. Thus, only *a posteriori* comments can be advanced on the observed pattern of results and results should be viewed with caution. At any rate, one may conjecture that the significance of the model would mark the contribution of item-based processing in a given task while its absence might indicate the preponderance of algorithm-based processing. In particular, individual learning parameters predicted speed in reading a text passage and the ability to read correctly and rapidly single words at the One-minute reading test. In this vein, the item-based processing allows ensuring an automatized reading and then a good reading speed, at least in a consistent orthography such as Italian (in which lexical processing is not necessary for ensuring accuracy, but it is indispensable for fluent reading). Furthermore, learning parameters also significantly predicted the accuracy in spelling a meaningful text passage and the total performance in the DDO-2 spelling test (in this case, this value includes the section of words with ambiguous transcription). By contrary, models failed to reach significance for the spelling of words without a 1:1 mapping (as well as for pseudowords) and approached significance in the case of regular words. Children with greater capacity to acquire instances showed better performance in spelling meaningful stimuli: this finding may suggest that regular words were generally spelled through the lexical procedure also in a consistent orthography such as Italian. Finally, learning parameters significantly predicted accuracy in reading numbers: the lexical retrieval of the number name is related to the ability to acquire instances. On the contrary, no relationship was found in the case of making written mathematical operations, probably due to an analytic application of computation procedures (at least at the age examined in the present study) instead of an automatic retrieval of the result.

We have noted in the introduction that very few studies have examined rate of acquisition in children with learning disorders, and most of these studies were focused on a single behavior (i.e., reading). The study by Nicolson and Fawcett (2000) is a notable exception as they examined the effect of learning new tasks as a function of an extended training. However, a direct comparison with this study is difficult. In particular, here we focused on a task that with practice could be solved by relying on instance learning; by contrast, the tasks used in the Nicolson and Fawcett’s (2000) study did not clearly call for learning of specific instances. Thus, apart from the use of different types of populations, the two studies appear to tackle different types of learning problems. A direct comparison is also difficult with studies investigating implicit learning of linguistic and non-linguistic regularities, both in typically developing children and in children with dyslexia (for a systematic review see Schmalz et al., 2017); for a meta-analysis see van Witteloostuijn et al., 2017). These studies use different experimental paradigms to present rule-governed situations (e.g., letter sequences or shape sequences or visual-motor rule-governed tasks) to participants who, unaware of the embedded rules, are requested to perform some sort of tasks (e.g., memorize or simply observe) in a first exposure phase and then, in a testing phase, are evaluated on their newly acquired knowledge related to the situation. However, to our knowledge these studies do not analyze the curves of acquisition but focus on group differences (e.g., readers with dyslexia vs. typically developing readers; adults vs. children) or paradigms/materials (e.g., linguistic vs. non-linguistic materials). Moreover, the relationship to literacy tasks is often speculated or inferred on the basis of a poor performance on implicit learning tasks in individuals with developmental dyslexia. However, Nigro et al. (2015) investigated the implicit learning ability in Spanish third grade typically developing children and found a significant correlation between the implicit learning task performance and the ability to spell words with unpredictable mapping, i.e., stimuli which require word specific knowledge to resolve the spelling inconsistencies. By contrast, they did not find any relationship with the word and non-word reading abilities and did not evaluate the mathematical domain. According to the authors, the implicit learning mechanism may play a role in the acquisition of lexical knowledge and thus, in writing proficiency. In spite of several methodological differences, the pattern of findings and the interpretation advanced by Nigro et al. (2015) presents a number of similarities to the present proposal.

Here, our main interest was in evaluating the hypothesis that a good learning ability, as assessed by better ability in consolidating instances, would act as a cross-domain predictor of performance. We feel that the present study well illustrates the complexities to pursue such a goal. First, the measure needs to be dynamic, i.e., it aims to capture the change in performance with practice not just the performance at one point in time. Second, in order to have a reliable measure of improvement one needs to refer to a model of learning. Indeed, simple measures of change such as the difference between the initial and final performance after training may not be ideal as this would be inevitably correlated with initial performance (for a discussion on the problems connected with difference scores see Capitani et al., 1999; Zoccolotti and Caracciolo, 2002). Finally, if the goal is to obtain a general measure of the ability to consolidate instances, the task should be as much as possible novel, that is independent from previously consolidated abilities.

These complexities indicate that it may actually be difficult to generate a clinically valid test to measure the ability to consolidate instances although this goal is certainly worth pursuing it. At the same time, it must be noted that failure to account for the role of experience may indeed be critical in fully understanding learning disorders. This point was persuasively made in a recent review of the research on dyslexia by Huettig et al. (2018). For example, these authors noted that most studies on illiterate subjects yielded results quite similar to studies on children with dyslexia. Accordingly, they raised the possibility that reading disorders may actually be a consequence of reduced and suboptimal reading experience. This does not necessarily indicate that learning disorders are merely epiphenomena of reduced practice. Rather, the analysis made by Huettig et al. (2018) underscores the difficulty in interpreting measures of performance taken only at a single point of time, as typical of standard clinical tests of reading (spelling or doing maths). Indeed, these measures express the joint effect of several different factors. First, individual performance may depend upon the individual ability in the behavior object of the test (such as reading, spelling or doing maths). However, second, individual performance at any point in time will also vary as an effect of the amount of practice on that task. Third, the performance will also express the ability of the individual to improve as an effect of practice. In other terms, the effect of practice may depend on its quantity but also on the individual capacity to take advantage from it. Within the instance theory of automatization, this individual dimension would specifically express as the capacity of consolidating instances. The important consequence of these multi-factorial influences is that there is no simple way to separate the effect of these components when examining a child under standard clinical conditions. Much to the contrary, it is likely that these components tend to interact to each other. Thus, it is well known that children who are not proficient in reading (spelling or doing maths) do not like to do these activities with the result that, all other things being equal, they tend to practice less.

Some limitations of the present study should be put forward. Based on the predictions of the multi-level model of learning skills, we originally planned to have measures for which individual learning rate would not be associated in all domains considered, i.e., reading, spelling, and maths. To this aim, in reading, we planned to use a pseudo-word reading task. However, due to problems during data collection, information on this specific task was not obtained in most children. Therefore, the prediction that the individual rate of learning would not predict non-word reading still needs to be tested to be able to fully appreciate the predictions of the model.

The model presented in Figure 1 aims to predict performance both in the typically developing range as well as in the impaired range (i.e., the well-known comorbidity among learning disorders). In the present study, as well as our previous one (Zoccolotti et al., 2020a, 2020b), we examined unselected populations of children. Therefore, before confirming the specific role of instance-based learning on the comorbidity of learning disorders, it will be crucial to directly test populations of children with different patterns of learning disabilities. This study is currently under way (although severely slowed down by the current pandemic). In particular, we predict that a low ability in consolidating instances will be particularly associated with some areas of processing, such as lexical activation, in the case of reading and spelling, and acquisition and retrieval of arithmetic facts, in the case of maths. In other words, this prediction is selective for some aspects of behavior, not the general ability of reading (spelling or doing maths). Extending data from unselected populations of children to the pathological range partly depends on the way learning disorders are conceptualized. In a line of thought, developmental disorders of reading (spelling and maths) are seen as the low end of a continuous distribution (e.g., Protopapas and Parrila, 2018). Alternatively, a body of literature has described qualitatively different patterns of impairments in reading as well as spelling and maths (for reviews see for example Geary, 2004; McCloskey and Rapp, 2017; Friedmann and Coltheart, 2018). It seems that focused research is needed to clarify this important point. We propose here that referring to an individual dimension of “ability to consolidate instances” may provide an interesting heuristic for studying the comorbidity across learning disorders.

Reviewing a large body of neurophysiological evidence, Keresztes et al. (2018) have proposed that the learning system has to balance the need of “*detecting regularities in the world through generalization* versus *encoding and remembering particular events and their details through mnemonic specificity.*” In our previous work (Zoccolotti et al., 2020a,b), we have proposed that the ability to use information from specific events, conceptualized as a dimension of “*ability to consolidate instances,*” is a general-purpose skill that may foster performance across domains. The present findings provide some support to this hypothesis since the learning rate on a novel task was selectively correlated with performance requiring acquired knowledge of individual items across reading, spelling, and maths tasks. While there is certainly a need for further work in this area, we propose that the procedure developed here may provide useful insights on the contribution of the role of automatization skills in the genesis of learning disorders.

## Data Availability Statement

The raw data supporting the conclusions of this article will be made available by the authors, without undue reservation.

## Ethics Statement

The studies involving human participants were reviewed and approved by Comitato Etico – Fondazione Santa Lucia, Rome, Italy. Written informed consent to participate in this study was provided by the participants’ legal guardian/next of kin.

## Author Contributions

CM, PA, MM, MT, and PZ contributed conception and design of the study. MT organized and supervised the database collection. CM and MM performed the statistical analyses. PZ wrote the first draft of the manuscript. All authors wrote sections of the manuscript, contributed to manuscript revision, read and approved the submitted version.

## Conflict of Interest

The authors declare that the research was conducted in the absence of any commercial or financial relationships that could be construed as a potential conflict of interest.

## Publisher’s Note

All claims expressed in this article are solely those of the authors and do not necessarily represent those of their affiliated organizations, or those of the publisher, the editors and the reviewers. Any product that may be evaluated in this article, or claim that may be made by its manufacturer, is not guaranteed or endorsed by the publisher.
